# Limit cycles with transient state dynamics in cyclic networks

**DOI:** 10.1186/1471-2202-16-S1-P89

**Published:** 2015-12-18

**Authors:** Bulcsú Sándor, Claudius Gros

**Affiliations:** 1 Institut für Theoretische Physik, Goethe Universität, Frankfurt am Main, 60438, Germany

## 

Changes in the transmission properties of synapses may influence actively the processing of information. This is in particular the case for the working memory, viz the temporary storage of information, which is thought to be mediated via short-term or transient synaptic plasticity effects [1]. The standard Tsodyks-Markram model [2] for short-term synaptic plasticity allows, in this context, the statistical investigation of spiking neural networks as well as the mean field analysis of rate encoding neural populations [3].

**Figure 1:**
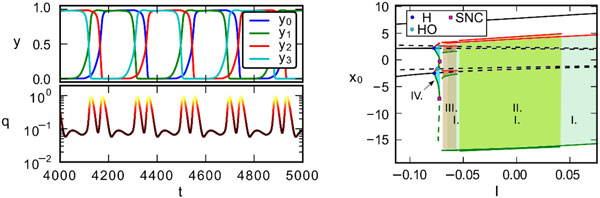
**The firing rates of the respective neural populations in the ring network, and the corresponding phase space speed q of the trajectory as a function of time (top and bottom plots of the left panel)**. The bifurcation diagram of the system as a function the external input I (right panel). The parameter intervals of four possible limit cycles classes denoted by Roman numerals I-IV are indicated by different green/blue shades. H points denote Hopf-bifurcation, HO is homoclinic bifurcation of saddles, SNC denotes saddle node bifurcation of cycles. The maximal/minimal amplitude in a limit cycle is denoted with red/green color. Stable (unstable) fixpoints and limit cycles are continuous (dashed) curves.

We implement a slightly modified version of the Tsodyks-Markram model for cyclic networks characterized by Mexican-hat type connectivity profiles for the synaptic weights. The system shows a surprisingly rich set of dynamical states, even for rings of only four neurons (or neural populations), such as transient state dynamics [4]. In this case one observes extended plateaus in the time series of the population firing rates, indicating well defined transient states (see left panel of Figure 1).

Going beyond the simple mean-field analysis, we present an in-depth study of the bifurcation diagram, as a function of several bifurcation parameters, such as the strength I of the external current or the integration time 1/Γ of the neural activity. Four classes of distinct limit cycles are found (see right panel of Figure 1), in addition to stable fixpoints and saddles. We note, that our results are not only important for an in-depth understanding of the network effects of working memory, but also indicate the possibility to construct central pattern generators using short-term synaptic plasticity.
